# Prevalence of Hypothyroidism and its Impact on Cognitive Function in Older Adults: A Cross Sectional Study

**DOI:** 10.1002/alz.091284

**Published:** 2025-01-09

**Authors:** Angeline Jessy S, Sandhya G, Monisha S, Thomas Gregor Issac

**Affiliations:** ^1^ Centre for Brain Research, Bangalore, Karnataka India; ^2^ Centre for Brain Research, Bangalore, karnataka India; ^3^ Centre for Brain Research, Indian Institute of Science, Bangalore, Karnataka India

## Abstract

**Background:**

Thyroid disorders is one of the most common endocrine disorders. It is estimated that 42 million people suffer from thyroid disorders in India. The imbalance in thyroid hormone levels can significantly impact cognitive health of older population. This study aims at emphasizing the prevalence of hypothyroidism and its association with cognition.

**Method:**

A cross‐sectional design, the study enrolled 1,181 participants from the TLSA cohort, focusing on individuals with complete data for sociodemographic characteristics and thyroid‐related blood parameters. Comprehensive clinical assessments, including medical history, physical examinations, and cognitive tests namely COGNITO battery (Computerized Assessment of Adult information processing), HMSE (Hindi Mental State Examination) and ACE‐III (Addenbrooke’s Cognition Examination) were conducted. Biochemical tests quantified plasma levels of thyroid‐stimulating hormones (TSH), triiodothyronine (T3), and thyroxine (T4). Participants were classified based on medication history and thyroid hormone levels.

**Result:**

The study identified a 17.69% prevalence of hypothyroidism, among which 6.22% overt and 93.78% subclinical cases. Females exhibited a significantly higher prevalence (p=0.043). People with hypothyroidism are more frequently diagnosed with mild cognitive impairment than the people with euthyroid (p=0.008). Patients managed suboptimally performed poorer in auditory attention (p<0.001) compared to those on adequate treatment. In the domain of visual attention, the inadequately treated group performed significantly worse than the adequately treated group (p=0.018)

**Conclusion:**

Previous studies suggest a direct association between hypothyroidism and cognitive impairment. This study concludes that hypothyroidism has a significant relationship with cognitive functioning in the attention domain in older adults. Hence, hypothyroidism should be treated as a priority to maintain cognition in an ageing population.

